# Influence of Network Structure on Glass Transition Temperature of Elastomers

**DOI:** 10.3390/ma9070607

**Published:** 2016-07-22

**Authors:** Katarzyna Bandzierz, Louis Reuvekamp, Jerzy Dryzek, Wilma Dierkes, Anke Blume, Dariusz Bielinski

**Affiliations:** 1 Institute of Polymer and Dye Technology, Lodz University of Technology, Lodz 90-924, Poland; dariusz.bielinski@p.lodz.pl; 2 Elastomer Technology & Engineering, University of Twente, Enschede 7500 AE, The Netherlands; l.a.e.m.reuvekamp@utwente.nl (L.R.); w.k.dierkes@utwente.nl (W.D.); a.blume@utwente.nl (A.B.); 3 Institute of Nuclear Physics, Polish Academy of Sciences, Krakow 31-342, Poland; jerzy.dryzek@ifj.edu.pl

**Keywords:** elastomer, rubber, curatives, crosslinks, crosslink density, crosslink structures, glass transition, free volumes

## Abstract

It is generally believed that only intermolecular, elastically-effective crosslinks influence elastomer properties. The role of the intramolecular modifications of the polymer chains is marginalized. The aim of our study was the characterization of the structural parameters of cured elastomers, and determination of their influence on the behavior of the polymer network. For this purpose, styrene-butadiene rubbers (SBR), cured with various curatives, such as DCP, TMTD, TBzTD, Vulcuren^®^, DPG/S_8_, CBS/S_8_, MBTS/S_8_ and ZDT/S_8_, were investigated. In every series of samples a broad range of crosslink density was obtained, in addition to diverse crosslink structures, as determined by equilibrium swelling and thiol-amine analysis. Differential scanning calorimetry (DSC) and dynamic mechanical analysis (DMA) were used to study the glass transition process, and positron annihilation lifetime spectroscopy (PALS) to investigate the size of the free volumes. For all samples, the values of the glass transition temperature (*T*_g_) increased with a rise in crosslink density. At the same time, the free volume size proportionally decreased. The changes in *T*_g_ and free volume size show significant differences between the series crosslinked with various curatives. These variations are explained on the basis of the curatives’ structure effect. Furthermore, basic structure-property relationships are provided. They enable the prediction of the effect of curatives on the structural parameters of the network, and some of the resulting properties. It is proved that the applied techniques—DSC, DMA, and PALS—can serve to provide information about the modifications to the polymer chains. Moreover, on the basis of the obtained results and considering the diversified curatives available nowadays, the usability of “part per hundred rubber” (phr) unit is questioned.

## 1. Introduction

**Figure 1:**
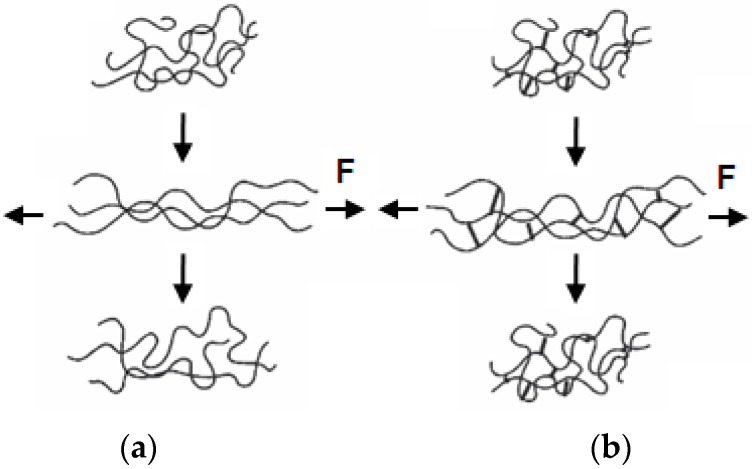
Performance of an elastomer under loading force and after its removal: (**a**) plastic deformation of an uncrosslinked elastomer; and (**b**) elastic recovery of a crosslinked elastomer.

Elastomers are a unique class of materials. They can withstand high elastic deformations, and recover to their original size and shape immediately after removal of the force. The majority of elastomers are rubbers. To exhibit elasticity, their chains have to be connected to each other by chemical bonds, called crosslinks. The crosslinks prevent untangling of the chains and sliding over each other under load. When crosslinked, intrinsically-viscoelastic elastomers behave in a “hyperelastic” way, and plastic deformations do not occur (Figure 1). 

Hence, crosslink density is a basic structural parameter which characterizes three-dimensional elastomer networks. It is defined as a number of crosslinks per unit volume in a polymer network [1]. Crosslink density influences, to a very high extent, various properties of elastomers [2]. Among them are tensile strength, mechanical modulus, hardness, swelling in solvents, glass transition temperature, and thermal stability. Crosslink density is usually analyzed by the equilibrium swelling of the elastomer in a thermodynamically-suitable solvent, and calculated on the basis of the Flory-Rehner equation [3]. Other methods of crosslink density determination refer to the Mooney-Rivlin model [4,5], or to nuclear magnetic resonance [6]. 

To introduce crosslinks into the elastomer, a curing process has to be carried out. It is conventionally performed with the use of curatives, which need high temperatures to be activated and start the chemical reactions for crosslinking. The curing process is, therefore, carried out at elevated temperatures (130–190 °C). The types of curatives are usually: (i) classical curing systems based on rhombic sulfur combined with an accelerator; (ii) sulfur donors; (iii) organic peroxides; or (iv) metal oxides. The curatives nowadays are abundant and characterize themselves with diverse structures, which increasingly often are very complex. Therefore, the curatives exhibit various and complicated reactivities, especially at elevated temperatures. This is particularly significant in case of sulfur compounds, rhombic sulfur and their mixtures. Hence, the transformation products and resulting crosslinks can be very diverse. 

The formed intermolecular elastically effective crosslinks, are usually classified as carbon-carbon (C–C) crosslinks and sulfidic crosslinks. The C–C crosslinks are created directly between the polymer chains. In contrast, the sulfidic crosslinks are made of a variable number of sulfur atoms. Depending on this number, sulfidic crosslinks are further subdivided into (i) monosulfidic (C–S–C); (ii) disulfidic (C–S_2_–C); and (iii) polysulfidic (C–S*_x_*–C, *x* ≥ 3) crosslinks. All of these crosslinks show varying structure and length, resulting in various characteristics. As a result, they provide different properties to the material. Therefore, it is generally assumed that after the crosslink density, the crosslink structure is the second most important parameter influencing the elastomer properties [7,8]. To characterize the various types of crosslinks, chemical analyses are used. The most commonly used is thiol-amine analysis [9], based on the selective cleavage of particular types of sulfidic crosslinks. This analysis was originally developed for natural rubber. Nowadays it is also often used for other types of elastomers [10,11].

**Figure 2:**
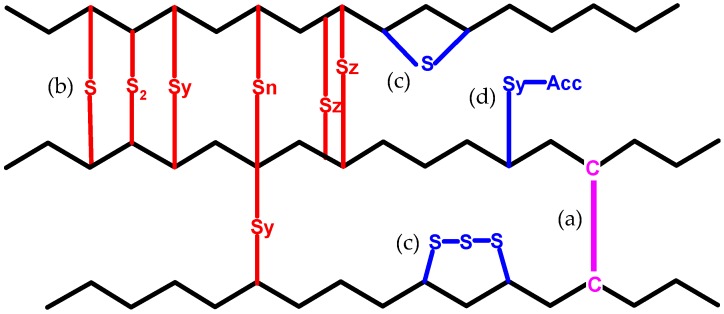
Structures formed as a result of curing intermolecular crosslinks: (**a**) carbon-carbon crosslinks; (**b**) sulfidic crosslinks, intramolecular modifications of the polymer chains: (**c**) cyclic sulfur structures; and (**d**) pendant groups.

Since the curing process is carried out at elevated temperatures, uncontrolled side reactions can also take place. Reactions of the curatives do not always lead to the formation of crosslinks to connect two polymer chains. Actually, part of the transformation products of the curatives can chemically attach to only one polymer chain. In this way, a non-elastic, intramolecular modification is formed, as schematically presented in Figure 2. Depending on the curatives, these modifications exhibit various structures. They can be in a configuration of pendant groups or cyclic structures, containing sulfur. Furthermore, since the curatives are introduced in amounts of several parts per hundred rubber (phr), those modifications can be abundant.

According to the literature [8,12], the elastomer properties are almost entirely dependent on the intermolecular crosslinks and their structure. At the same time, the presence of modifications to the polymer chains is barely mentioned. If these modifications are discussed, it is stated that they do not influence the material properties, or only to a limited extent. As a consequence, much attention is paid to the analysis of solely the crosslink density and their structures. In fact, there are no reliable direct methods to identify and quantify the modifications of the chains formed by the curatives. This causes difficulties in investigating these structures. The reactivity of the curatives can be very complex; therefore, it is a far-reaching simplification to consider the curing process only as formation of intermolecular crosslinks.

The present research discusses the unrecognized role of the elastomer curatives. It shows their influence on the network parameters and resulting properties. Due to the increasing number of curatives available nowadays, there arises the need for comprehensive research on their role in the curing process. For this purpose, elastomers cured with various types of curatives were prepared. The structural parameters, such as crosslink density and crosslink structure were characterized, using chemical methods—equilibrium swelling and thiol-amine analysis. The behavior of the elastomers was investigated via monitoring the glass transition process. Two microscale techniques were used for this: differential scanning calorimetry (DSC) and dynamic mechanical analysis (DMA). The influence of the various curatives on the size of the free volumes and molecular structure was characterized by positron annihilation lifetime spectroscopy (PALS). The different results between the samples are discussed. They are explained on the basis of the curatives’ structures and modifications of the chains formed by them.

## 2. Experimental

### 2.1. Materials

The polymer used in this study was a cold emulsion styrene-butadiene rubber (E-SBR) KER 1500, with 23.5% of bound styrene, produced by Synthos (Oswiecim, Poland). The ratio of styrene to butadiene units is therefore one to six or seven. The chemical structure of butadiene consists of 15% 1,2-vinyl, 10% 1,4-cis, and 75% 1,4-trans. The molecular weight of the SBR was *M*_n_ = 151,000 g/mol, *M*_w_ = 425,000 g/mol, and dispersity 2.8, as reported by Synthos. The Mooney viscosity was ML (1+4)100 °C = 50. Zinc oxide and stearic acid were obtained from Arlanxeo (Maastricht, The Netherlands). The curatives used are listed and characterized in Table 1. Acetone used for extraction, and toluene used for crosslink density analysis were of analytical grade, obtained from Assink Chemie (Enschede, The Netherlands). The chemicals for the thiol-amine probes: piperidine (99%), 2-propanethiol (≥98%), and 1-dodecanethiol (≥98%) were provided by Sigma-Aldrich (St. Louis, MO, USA).

### 2.2. Sample Preparation

#### 2.2.1. Mixing

The samples were mixed in two steps. The first step was performed in an internal micromixer Brabender Plasti-Corder (Duisburg, Germany), with a mixing chamber of 75 mL. The mixing procedure employed was first plasticizing the E-SBR for one minute. After that, the 5 phr of ZnO and 1 phr of stearic acid were added. The total mixing time was 5 min, and the mixing temperature did not exceed 50 °C. The first mixing step was immediately followed by a second step, carried out on a David Bridge (Rochdale, UK) two-roll open mixing mill. After one minute of mixing on the two-roll mill, the curatives were added. In case of accelerated sulfur curing (series: DPG/S_8_, CBS/S_8_, MBTS/S_8_, ZDT/S_8_), the accelerators were firstly incorporated. After another 3 min, the sulfur was added. The compounds were homogenized for 10 min, while the temperature did not exceed 40 °C. The compositions of the mixtures are given in Table 2. 

#### 2.2.2. Curing

After addition of the curing agents, the compounds curing properties were determined with an RPA2000 rubber process analyzer from Alpha Technologies (Bellingham, WA, USA). The increase in torque at 160 °C, 0.833 Hz and 2.79% strain was measured over a time period of 60 min. The optimal cure time (τ_95_) of the rubber compounds was determined. Rubber sheets with a thickness of 2 mm were cured in an electrically-heated Wickert laboratory press WLP 1600/5*4/3 (Landau, Germany) under 100 bar for the optimal cure time. The curing temperature for the S_8_ series was 180 °C, whereas for all of the other series it was 160 °C. The uncrosslinked reference (Ref.) was prepared by compression molding at a temperature of 160 °C for 10 min. 

### 2.3. Extraction with Acetone

To remove low molecular, non-polymer substances, the cured samples were extracted with acetone in a Soxhlet apparatus for 72 h. This was followed by drying them to a constant weight at 55 °C in a Heraeus (Hanau, Germany) oven with an air circulation system. The acetone-extracted samples were used for crosslink density analysis and DSC glass transition studies.

### 2.4. Crosslink Density Analysis

To analyze the crosslink density, samples of approx. 0.05 g were swollen to equilibrium in toluene for four days at room temperature (23 ± 1 °C). This was followed by drying the samples to a constant weight for four days at 60 °C in a Heraeus (Hanau, Germany) oven with an air circulation system. The crosslink density was calculated on basis of the Flory-Rehner equation [3]:

(1) $$V=-\frac{\ln\left(1-V_{r\;\;}\right)+V_{r\;\;}+\chi V_{r}^{2}}{V_{0}\;\left(\;{V_{r}}^{\frac{1}{3}}-\;\frac{2V_{r}}{f}\right)}$$

where:

*ν*—crosslink density per unit volume (mol/cm^3^);
*V_r_*—volume fraction of rubber in a swollen sample (-);
*V*_0_—solvent molar volume (for toluene: *V*_0_ = 106.9 cm^3^/mol);
*f*—functionality of crosslinks (*f* = 4, assuming the formation of tetra-functional crosslinks);
χ—Flory-Huggins rubber-solvent interaction parameter (for investigated SBR-toluene system: χ = 0.378 [14]).

The given crosslink density values are the average of four specimens of every sample. The relative standard deviation is approx. 3.0% on average. 

### 2.5. Crosslink Structure Analysis

The structure of the crosslinks was determined by thiol-amine analysis [9]. This is based on selective cleavage of particular types of sulfidic crosslinks by two sets of thiol-amine chemical probes. Polysulfidic crosslinks were cleaved by the “soft” probe, composed of 2-propanethiol (0.4 M) and piperidine (0.4 M) in toluene. This analysis was run for 2 h under nitrogen gas atmosphere in a closed glass vial, using rubber samples pre-swollen in toluene for 12 h. Polysulfidic and disulfidic crosslinks were cleaved by the “hard” probe, which composition was 1-dodecanethiol (1.0 M) in piperidine. This analysis was run for 24 h under nitrogen gas atmosphere in a closed glass vial. After the thiol-amine analyses, the samples were removed from the probes immediately. The probe residues were removed from the samples by washing them at least five times with toluene for 30 min at room temperature. After this, the remaining crosslink density was analyzed by equilibrium swelling in toluene and calculated on the basis of the Flory-Rehner equation (according to the procedure described in Section 2.4.).

### 2.6. Buoyancy Method

To determine the density of the uncrosslinked reference and the cured samples, a buoyancy method was used. The measurements were carried out in ethanol at a temperature of 23 °C. Analytical balance Radwag (Radom, Poland), equipped with a standard kit for density determination, was used. The given density is an average of six measurements. The relative standard deviation of the experiment was approx. 2%.

### 2.7. Differential Scanning Calorimetry (DSC)

Differential scanning calorimetry (DSC) measurements were performed with use of a DSC Polyma 214 calorimeter from Netzsch-Geratebau (Selb, Germany). The samples of approx. 10 mg were placed in standard aluminum pans with pierced lids. The measurements were carried out in nitrogen atmosphere as follows: (i) cooled at a rate of 10 °C/min from 30 to −110 °C; (ii)stabilized at −110 °C for 3 min; and (iii) heated at a rate of 10 °C/min from −110 to 30 °C. Prior extraction of the samples with acetone served to obtain smoother thermograms, without influencing the *T*_g_ values. A thermal history effect was checked by recording three measurement cycles for a representative set of samples. The same *T*_g_ values were obtained, which showed that the measurements were not sensitive to thermal history. The data shown here were, therefore, collected during the first heating scan. The values of glass transition temperature were taken as the midpoints of the heat capacity change during the heating step.

### 2.8. Dynamic Mechanical Analysis (DMA)

Dynamic mechanical measurements were performed with use of a dynamic-mechanical analyzer DMA Viscoanalyseur VA2000 from Metravib (Limonest, France), in tension mode. The test frequency was fixed at 10 Hz, and the strain at 0.1%, which was within the linear viscoelastic region of the studied samples. The dimensions of the test bar specimens were 40 × 5 × 2 mm^3^. Acquisition of the data was done using Dynatest 6.83 software. The measurements were carried out with a temperature sweep in the range of −80 °C to 80 °C. In the temperature range of the *E*’’ and tan delta changes, the temperature interval was 1 °C. This allowed establishing an accurate determination of the *T*_g_ values. In the rest of the measurement, the interval was 5 °C. The initial stabilization time at −80 °C was set to 15 min, whereas for other measurement points it was 3 min.

### 2.9. Positron Annihilation Lifetime Spectroscopy (PALS)

The measurements of the positron lifetime spectra of the samples were performed using a *fast–fast* positron lifetime spectrometer with BaF_2_ scintillators. The time resolution (FWHM) of the lifetime spectrometer was approx. 250 ps. The positron source, ^22^Na enveloped in 7 µm Kapton foil, was located between the surfaces of two identical samples. This sandwich was positioned in front of the scintillation detectors of the spectrometer. The positron lifetime spectrum was measured for 24 h to obtain more than 2 × 10^6^ counts in the spectrum. Deconvolution of each spectrum was performed assuming three lifetime components. This was sufficient to obtain a satisfactory χ^2^ value close to unity. The source component was also taken into account during the deconvolution procedure. The error bar of the free volume radius is expressed as the standard deviation of the measurement.

## 3. Results and Discussion

### 3.1. Crosslink Density and Crosslink Structures

**Figure 3:**
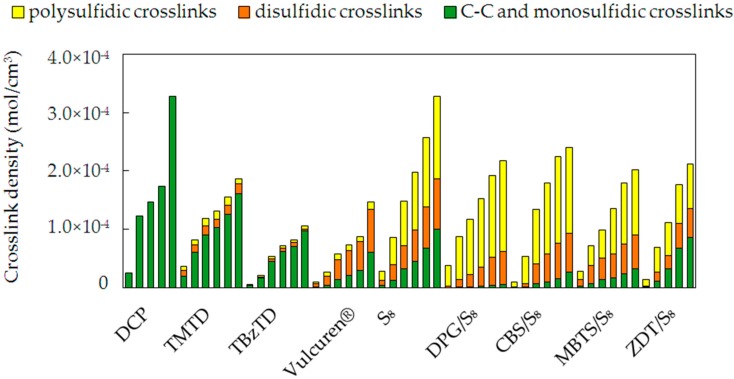
Crosslink density and crosslink structures formed during the curing process with use of various curatives. The columns in each series correspond to the samples with an increasing amount of curatives, as listed in Table 2.

In all studied series of samples, an increasing amount of the added curatives resulted in the formation of a higher crosslink density. This is due to more chemical species, which were involved in the formation of chemical bonds between the polymer chains. In Figure 3, the crosslink density and participation of the various crosslinks are presented.

Curing with various curatives resulted in the formation of diversified crosslink structures. Use of organic peroxide, i.e., DCP, led to the formation of solely carbon-carbon crosslinks. During the curing process with pure TMTD, mainly C–C and monosulfidic crosslinks were formed. TBzTD acted upon curing in a similar way to TMTD and produced short crosslinks, mainly monosulfidic. TBzTD also belongs to the class of thiurams and has benzyl groups instead of the methyl groups present in a TMTD molecule. Use of Vulcuren^®^ resulted in the formation of longer crosslinks, with major participation of the disulfidic ones. The structure of Vulcuren^®^ is comparable with TBzTD and, additionally, has a six-carbon spacer between two disulfide groups. However, the crosslink structure of TBzTD- and Vulcuren^®^-cured samples differs noticeably. To produce a sufficient crosslink density, the amount of incorporated TBzTD and Vulcuren^®^ had to be very high. This is due to the high molecular weight of these molecules. Use of pure sulfur S_8_ led to the formation of predominantly polysulfidic and disulfidic crosslinks. This is caused by the fact that sulfur, alone, reacts slowly and mainly develops long crosslinks. In case of accelerated sulfur curing systems, DPG/S_8_, CBS/S_8_, MBTS/S_8_ and ZDT/S_8_, the ratio in phr between the accelerator and sulfur in all series was 1. This classifies them as semi-efficient curing systems. Accelerators, such as MBTS, DPG, and CBS, combined with rhombic sulfur S_8_, led to the formation of mainly polysulfidic and disulfidic crosslinks. The share of short crosslinks was very low for these samples. Curing with ZDT/S_8_ resulted in the formation of approximately equal participation of short C–C and monosulfidic crosslinks, medium-length disulfidic, and longer polysulfidic crosslinks.

### 3.2. Effect of Curing on the Glass Transition Temperature

#### 3.2.1. Glass Transition Temperature Determined by Differential Scanning Calorimetry

**Figure 4:**
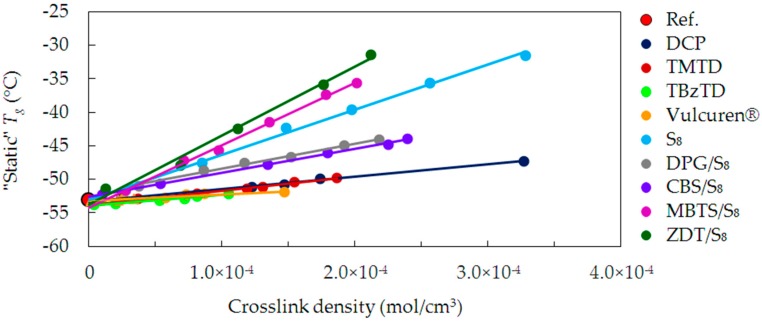
“Static” *T*_g_ as a function of crosslink density for the uncrosslinked reference and all cured series of samples.

During the glass transition process, one of the properties that exhibit abrupt change is the heat capacity. The DSC technique serves to investigate this, and determine the glass transition temperature (*T*_g_). The *T*_g_ measured in this way is sometimes referred to as “calorimetric”. Due to the fact that the sample is not exposed to external deformations, it is also called “static” *T*_g_. Figure 4 shows changes in *T*_g_ as a function of crosslink density, for the uncrosslinked reference and the series of samples cured with various curatives.

For the uncrosslinked reference (Ref.), the “static” *T*_g_ is approx. −53 °C. It has no chemical crosslinks and the chains interact with each other via weak van der Waals forces. Hence, the value of *T*_g_ comes from the microstructure of SBR—its building blocks, the relative participation of styrene and butadiene, and molecular weight. 

Compared to the reference, for all cured samples the *T*_g_ values linearly increase with a rise in crosslink density. As a result of the crosslinking, polymer chains were united with short, strong covalent bonds. This made the cured material more compact and stiffer. Crosslinks are topological constraints, which hinder segmental mobility of the chains. The denser the network is, the shorter the molecular segments between crosslinks are. The increase in the crosslink density is, therefore, followed by a rise in *T*_g_. This is caused by higher thermal energy required to enable the molecular mobility of the polymer segments. 

Actually, it is a well-known phenomenon [15,16] that the presence of crosslinks causes an increase in *T*_g_. Fox and Loshaek [17] derived equations, predicting linear dependency of *T*_g_ on the crosslink density. This prediction concerned polymers with a fixed composition and having a sufficiently low crosslink density. It assumed failure of linearity at higher crosslink densities. In the present research, good linearity is evident in the whole range of crosslink density for all series cured with various curatives. However, trends for particular series of samples show significant differences. They come from other structural factors than the total crosslink density. Figure 4 shows that the increase in *T*_g_ is the lowest for the series cured with single-component curatives, such as DCP, TMTD, TBzTD, and Vulcuren^®^. The increase in *T*_g_ is higher in case of CBS/S_8_, DPG/S_8_ and pure sulfur S_8_ series. The largest change is for MTBS/S_8_ and ZDT/S_8_. To explain differences between the series, their structural features have to be discussed in detail.

As a result of curing with an organic peroxide DCP, C-C crosslinks were formed. They are short and rigid and, hence, provide good elasticity and high stiffness to the material. It would be logical to assume that due to these characteristics they will cause a large increase in *T*_g_. Actually, the situation is the opposite: an increase in the crosslink density caused little changes in *T*_g_. Additionally, the DCP series is very much in line with the dependency for the TMTD series. Curing with TMTD also produced C–C and monosulfidic crosslinks. On the basis of these identical trends, a few remarks can be made. Firstly, DCP and TMTD led to formation of very short and undiversified crosslinks. Secondly, DCP and TMTD are curatives of low molecular weights and uncomplicated structures. Therefore, complex rearrangement reactions of these curatives were not possible. This made their reactivity at elevated temperature uncomplicated. Due to the low molecular weight, a part of the decomposition products possibly volatized and liberated from the system. Further, in addition to the formation of intermolecular crosslinks, the DCP and TMTD curatives modified the polymer chains by grafting on them. However, due to their small size, the DCP and TMTD did not stiffen the polymer chains in this way. As a result, the chains remained flexible and mobile. Due to all these factors, an increase in crosslink density caused very low rise in *T*_g_.

For TBzTD and Vulcuren^®^ series, the change of *T*_g_ with an increase in the crosslink density is also small. This might be caused by the presence of benzyl groups in the structure of those curatives. These groups are also present in the SBR structure as styrene units, which makes the TBzTD and Vulcuren^®^ molecules alike to the polymer matrix. In spite of their large amount and bulkiness, they led to a minor change in heat capacity. Therefore, the increase in *T*_g_ was small. Additionally, despite the difference in the crosslink structure of TBzTD and Vulcuren^®^ samples, their *T*_g_ changes are similar. This suggest that the crosslink structure is of minor importance.

Curing with rhombic sulfur S_8_ led to a large increase in *T*_g_ as a function of the crosslink density. This increase is evidently higher than for the above discussed single-component curatives. Curing with pure sulfur is inefficient—it has a slow curing rate. Moreover, it produces a large number of polysulfidic and intramolecular cyclic structures [18,19]. These numerous modifications restricted the free rotation possibilities of a single polymer chain. In this way, they hindered the mobility of the chains and considerably stiffened them. As a result, the *T*_g_ significantly increased.

In case of accelerated sulfur curing, the increase in *T*_g_ as a function of crosslink density is strongly dependent on the type of accelerator used. The increase for the DPG/S_8_ and CBS/S_8_ series is lower, when compared to the sulfur cured samples (S_8_). This can arise from two facts. Firstly, when the accelerator is combined with sulfur, the formation of intermolecular crosslinks is more efficient. It leads to lower quantity of intramolecular modifications of the polymer chains. Secondly, the DPG and CBS molecules have a low molecular weight (211.26 g/mol and 264.41 g/mol, respectively) and are small in size. Hence, the pendant groups of the accelerator fragments are not bulky. Therefore, they do not hinder the free rotation of the macromolecular segments to such a high degree like the cyclic sulfur structures do. These two effects can contribute to the observed trend lines as shown in Figure 4. Additionally, DPG/S_8_ and CBS/S_8_ series exhibit very alike dependencies, because the accelerators are similar in molecular weight and size. As mentioned before, the ratio of accelerator to sulfur, expressed in “phr” unit, was 1. Considering the molecular weight of the curatives, and calculating their amount in moles, this ratio is slightly different. For DPG/S_8_ it equals 1.18, whereas for CBS/S_8_ it is 0.92 (Table 3).

For the MBTS/S_8_ series, the increase in *T*_g_ is higher than for the series cured with pure sulfur. This is due to the structure of the MBTS molecule. It has a high molecular weight (332.49 g/mol) and aromatic heterocyclic moieties, which exhibit high stiffness. Fragments of the accelerator, together with sulfur, grafted onto the polymer chains during the curing process. Due to the bulkiness and inflexibility of these structures, this modification caused considerable stiffening of the polymer chains. Consequently, it was followed by a large increase in *T*_g_. The ratio between the number of moles of MBTS and S_8_ equals 0.73. It is lower than for the DPG/S_8_ and CBS/S_8_ series, in which the accelerator molecules are smaller. Furthermore, the MBTS/S_8_ samples have a similar crosslink structure compared to the DPG/S_8_ and CBS/S_8_ samples. However, the *T*_g_ values differ between those series. This proves that not the crosslink structure, but the accelerator type influences the behavior of the polymer chains.

In case of the samples cured with ZDT/S_8_, the increase in *T*_g_ with rise in crosslink density is very large. It is much higher than for the S_8_ series, and the greatest from all the studied curing systems. This effect is due to the ZDT molecular weight of 772.47 g/mol, which is the largest of all used accelerators. The ZDT structure is complex and has the bulky alkyl moieties. Similarly to the above discussed systems, modifications of the chains were formed by the curatives during the curing process. Due to their bulkiness, the mobility of the macromolecular segments was limited to a high extent. This caused a significant increase in the *T*_g_. Furthermore, ZDT is a polar molecule, which also possibly contributed to this rise. The ZDT fragments bound to the chains could interact with other similar fragments via intermolecular specific interactions. This additionally restricted the mobility of the polymer chains. As a result of curing, the crosslink structure developed in ZDT/S_8_ and sulfur S_8_ samples was similar. However, the *T*_g_ values of these two series differ significantly. The comparable crosslink structure does not result in the same behavior of the polymer chains. It is the various types of intramolecular modifications, which explain the differences. In case of ZDT/S_8_, the bulky fragments of accelerator, together with sulfur, grafted of the polymer chains. In case of S_8_ series, the modifications were in a form of cyclic structures and pendant groups composed of sulfur. The combined ZDT/S_8_ curatives stiffened the polymer chains to a greater extent than solely sulfur S_8_. 

As shown in Table 3, one-to-one ratio between ZDT and S_8_ is as low as 0.17, when their number of moles is considered. This means that the number of ZDT molecules was in a very small amount in relation to the molecules of rhombic sulfur. In fact, “phr” is a simplified unit, used to easily calculate the amount of various additives in a rubber mix formulation on a weight basis. Due to its universality, it was widely accepted by rubber technologists and research scientists. However, as the above described examples show, the “phr” unit used to calculate the amount of curatives can be misleading. Curatives can exhibit diversified structure and molecular weight and this unit does not consider these important variables. Therefore, in case of substances, such as curatives, it is non-informational and indirect. To overcome this problem, the amount of curatives should be expressed in “mol” unit. It considers the molecular weight, so that the number of reacting molecules is clearly expressed.

#### 3.2.2. Glass Transition Process Monitored by Dynamic Mechanical Analysis

**Figure 5:**
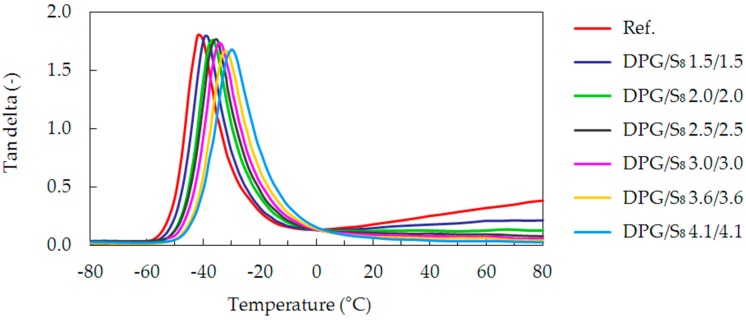
Tan delta as a function of temperature for the uncrosslinked reference and the DPG/S_8_ cured series of samples.

DMA tests were performed to study microscale changes in molecular mobility, as a response to externally applied mechanical deformations on the samples. In this way the “dynamic” *T*_g_ values were determined by the maximum of the tan delta peak. Figure 5 presents exemplary data from the DMA measurements, represented by the DPG/S_8_ series. They show the tan delta plotted versus the temperature for different crosslink densities.

**Figure 6:**
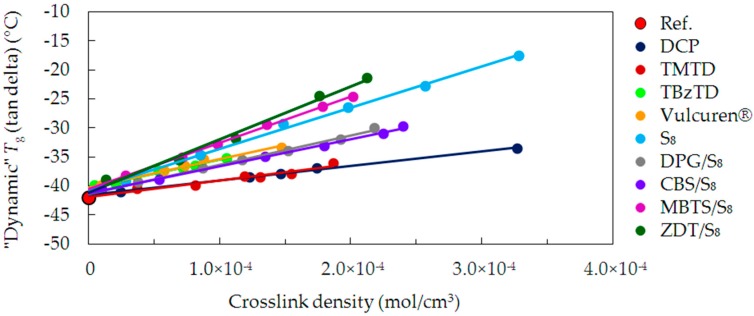
“Dynamic” *T*_g_, determined by a maximum of the tan delta peak, as a function of the crosslink density for the uncrosslinked reference and all cured series of samples.

Figure 6 depictures the “dynamic” *T*_g_ values as a function of the crosslink density, for all of the investigated samples. The “dynamic“ *T*_g_ shifts to higher values with increasing crosslink density when compared to the uncrosslinked reference. These changes of *T*_g_ are related to the chains’ relaxation. The “dynamic” *T*_g_ values show significant differences between the series cured with various curatives, as already observed in case of the “static” *T*_g_.

For the uncrosslinked reference, the “dynamic” *T*_g_ is approx. −42 °C. It is significantly larger than the “static” *T*_g_ (−53 °C). Such a difference is caused by frequency of 10 Hz in DMA measurement. Due to this, the “dynamic” *T*_g_ shifted to a higher temperature. 

In case of the cured samples, the lowest increase in the “dynamic” *T*_g_ is for the DCP and TMTD series. This corresponds to the dependencies in the “static” *T*_g_. However, for the TBzTD and Vulcuren^®^ samples, the increase in “dynamic” *T*_g_ is significantly larger than in case of the “static” *T*_g_. This effect arises from differences in the measurement techniques of DSC and DMA. Both in DMA and DSC measurements, molecular motions of the polymer segments begin at a temperature associated with the glass transition. The polymer segments are restricted by the crosslinks and modifications. In DSC, the sample is static, whereas in DMA it is deformed. Thus, in DMA, the mobility of the chains is a response to the deformations of a particular frequency. As a result, segments of the chains undergo conformational rearrangements. It minimizes localized stress, as it is possible on an experimental time scale. Curing with TBzTD and Vulcuren^®^ led to the formation of stiff and bulky structures. They require high thermal energy to enable molecular mobility under dynamic conditions. The trend lines for the TBzTD and Vulcuren^®^ series are similar to those of DPG/S_8_ and CBS/S_8_. The “dynamic” *T*_g_ of the sulfur cured samples (S_8_), show a further increase in *T*_g_. In case of MBTS/S_8_ cured samples, a higher increase in *T*_g_ is observed. This is due to the inflexible and bulky moieties of MBTS, combined with sulfur, causing a significant stiffening of the polymer chains. Finally, the most evident increase in *T*_g_ was observed for ZDT/S_8_. In this series the accelerator molecules are the largest and cause the highest stiffening of the polymer chains.

**Figure 7:**
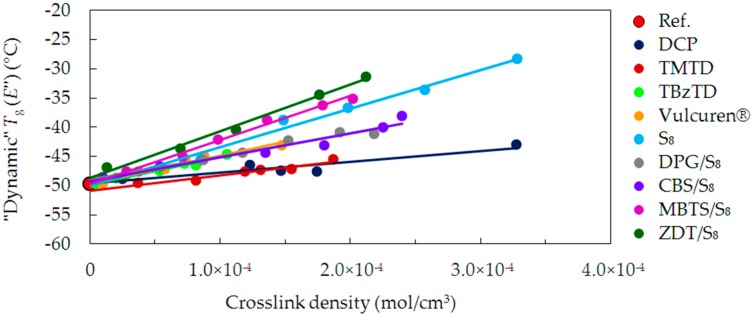
“Dynamic” *T*_g_, determined by a maximum of *E*’’, as a function of crosslink density for the uncrosslinked reference and all cured series of samples.

The “dynamic” glass transition process is sometimes also ascribed to the maximum of the loss modulus (*E*’’) peak. Figure 7 presents the *T*_g_ values determined from *E*’’. The *T*_g_ for the uncrosslinked reference is approx. −50 °C. All cured series of samples show a tendency corresponding to the tan delta “dynamic” *T*_g_. Differences between the maxima of the tan delta and *E*’’ peaks are within the range of 8–11 °C. The values of *E*’’ “dynamic” *T*_g_ are close to values of “static” *T*_g_. 

**Figure 8:**
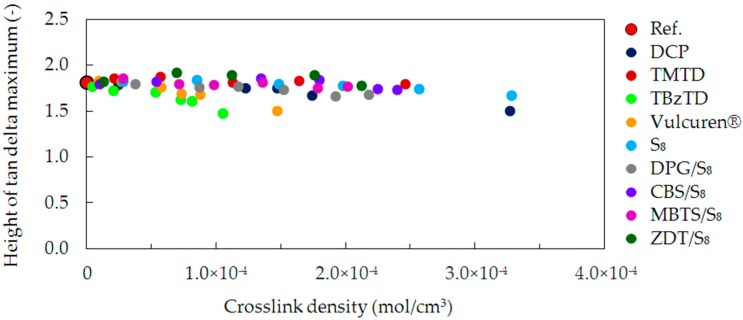
Height of tan delta peak as a function of crosslink density for the uncrosslinked reference and all cured series of samples.

To evaluate the elastic properties of the studied samples, the height of the tan delta peaks was correlated with the crosslink density (Figure 8). The largest value was observed for the uncrosslinked reference, due to its high plasticity. In case of the cured samples, the increase in the crosslink density was followed by a decrease in the tan delta height. The elastic properties were, therefore, improved. This effect is caused by the higher number of crosslinks connecting the polymer chains. The crosslinks serve to efficiently recover to the unstrained spatial network. The trend is the strongest for the TBzTD and Vulcuren^®^ cured samples.

**Figure 9:**
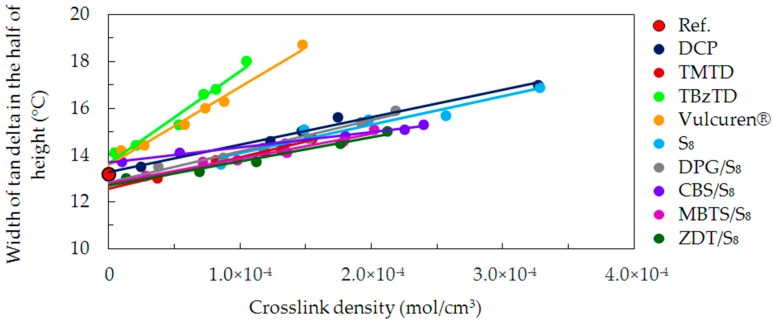
Width of tan delta peak in the half of height, as a function of crosslink density for the uncrosslinked reference and all cured series of samples.

The range of temperatures, in which the molecular mobility begins, was determined. For this, the width of the tan delta peak in the half of its height was calculated. Figure 9 shows values of this parameter, correlated with the crosslink density. An increase in the crosslink density resulted in a broadening of the tan delta peak. This is probably caused by more densely located structures on the chains formed by curatives. Additionally, the number and size of heterogenic microregions increased. These structures relaxed over a broader range of temperature. Changes in width of the tan delta peak are similar for most of the investigated series of samples, such as DCP, TMTD, S_8_, DPG/S_8_, CBS/S_8_, MBTS/S_8_, and ZDT/S_8_. However, in case of the TBzTD or Vulcuren^®^ samples, this increase is much higher. This can be caused by the large amounts of TBzTD and Vulcuren^®^ present in the samples. Consequently, the molecular movements during the glass transition process occurred over a broad range of temperature.

**Figure 10:**
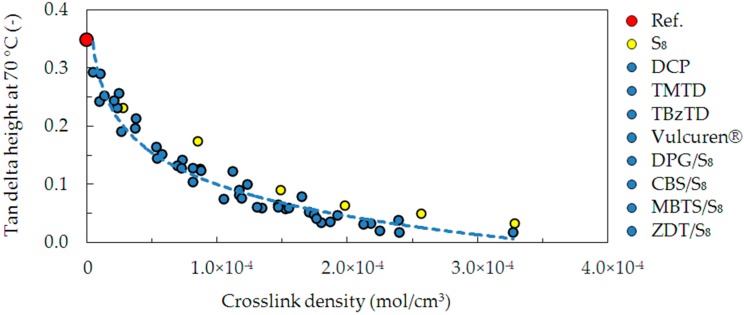
Dependence between height of tan delta at 70 °C and the crosslink density for the uncrosslinked reference and all cured series of samples.

The damping properties at elevated temperature, i.e., 70 °C, were evaluated by determination of the tan delta. Figure 10 presents these values, correlated with the crosslink density. The dependence shows that an increase in the crosslink density resulted in a large decrease in damping. This is due to an increase in the number of crosslinks, which provide elastic response and lower dissipation of energy. This dependence is similar in most of the investigated series of samples: DCP, TMTD, TBzTD, Vulcuren^®^, DPG/S_8_, CBS/S_8_, MBTS/S_8_, and ZDT/S_8_. However, in the case of samples cured with pure sulfur S_8_, the investigated ability to dissipate energy is slightly larger. This can be attributed to the fact that in addition to crosslinking, pure sulfur produced numerous modifications of the polymer chains. They are not elastically effective, hence the slightly larger dissipation of the energy. Additionally, the S_8_ series was cured at 180 °C, whereas all the other series at 160 °C. The higher temperature could result in increased chain scission in the sulfur-cured samples. This could contribute to different behavior of the S_8_ cured samples.

As discussed above, the presence of crosslinks caused an increase in the *T*_g_. This effect considerably differs between the samples cured with the various curatives. Therefore, other structural parameters than the total crosslink density contribute greatly to the changes in *T*_g_. The obtained results show that the crosslink structure does not explain them. It can be concluded that this parameter is not of a crucial importance. The differences between the *T*_g_ values of particular samples are ascribed to the presence of the curatives. They chemically attach to the polymer chains and form intramolecular modifications. If the curatives substances are small in size, i.e., have a low molecular weight, they do not affect the flexibility of the polymer chains. However, curatives having a high molecular weight, and bulky inflexible moieties, stiffen the polymer chains. It causes significant decrease of mobility of the chains, especially in vinyl polymers [20], such as SBR. This rotational restriction of these macromolecules influences the glass transition process and increases the *T*_g_ values considerably. Furthermore, the chemical structures of the curatives are usually very different from the structure of the basic polymers. It can, therefore, be considered that as a result of curing, a copolymer is formed, as already proposed by Fox and Loshaek [17]. In this way, curatives grafted on the chains can be considered as functional groups, which modify the polymer chains. The *T*_g_ of the cured system is not only dependent on the crosslinks themselves. Additionally, the average composition of the system plays a role. 

### 3.3. Effect of Curing on the Molecular Structure and Packing of the Polymer Chains

#### 3.3.1. Density Determined by Buoyancy Method

**Figure 11:**
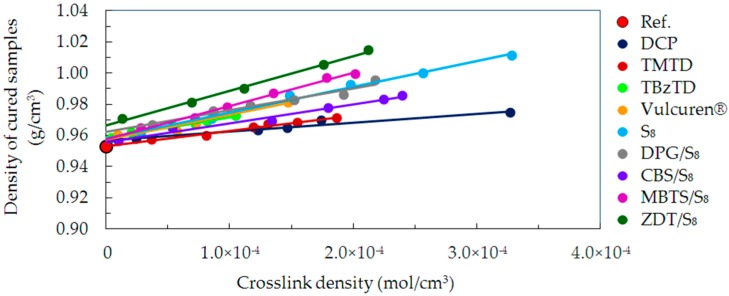
Density of samples as a function of crosslink density for the uncrosslinked reference and all cured series of samples.

The density of the samples cured with various curatives was measured by buoyancy method. Figure 11 shows the density values as a function of crosslink density.

The presented dependence indicates the density caused by structural packing of the cured polymer chains. The uncrosslinked reference characterized itself with the lowest density. For all series of samples, the density linearly increased together with an increase in the crosslink density. The change in density varied for particular series of samples. The least dense packing was observed for DCP and TMTD series, increasing for CBS/S_8_, then DPG/S_8_, Vulcuren^®^ and TBzTD. Further rise was observed for series cured with pure sulfur S_8_, followed by MBTS/S_8_. Finally, the highest increase was for the ZDT/S_8_ cured samples. 

The density measurements indicate that the material becomes more compact as a result of curing. Furthermore, structures formed by curatives locate and pack tightly in areas between the polymer chains. In general, the larger the molecules of the curatives are, the denser the packing of polymer chains with crosslinks and grafted modifications is. The density values show a direct correlation with *T*_g_ values, as investigated by DMA (Section 3.2.2).

#### 3.3.2. Free Volume Size Determined by Positron Annihilation Lifetime Spectroscopy

**Figure 12:**
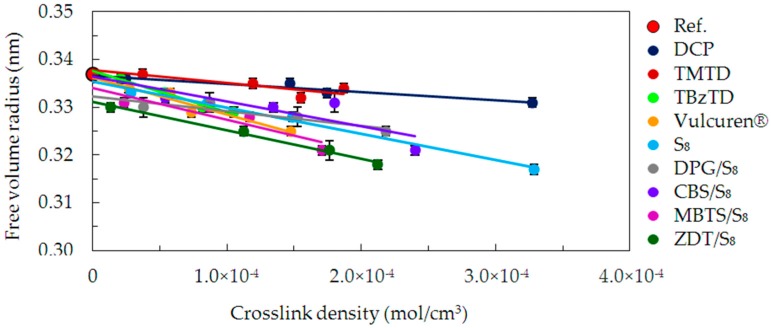
Size of free volumes as a function of crosslink density for the uncrosslinked reference and all cured series of samples.

PALS is a very capable experimental tool to study the structure of free volumes in polymers and related materials. In this context, free volume is defined as an area of significantly decreased electron density. Therefore, it is not occupied by any molecular structure. In the PALS spectrum of the polymers, the value of the longest lifetime component correlates with the size of the free volume. A direct relation between the value of this lifetime component and a radius of the free volume is given by the Tao-Eldrup equation [21,22]. This dependence is for a spherical symmetry of the free volume. Room-temperature PALS measurements were carried out to evaluate the effect of curing on the size of free volumes. Figure 12 shows changes in free volume size, expressed as a radius, as a function of crosslink density. 

The size of free volume is the largest for the uncrosslinked reference. For all cured samples, an increase in the crosslink density resulted in a reduction of the free volume size. Moreover, the degree of this change is strongly dependent on the type of curatives used. The lowest decrease in free volume size, show the samples cured with DCP and TMTD—curatives of small size molecules. A visibly larger decrease in free volume size is observed for CBS/S_8_ and DPG/S_8_ series, in which both accelerator and sulfur are present. Therefore, the modifications with bulky side groups grafted on the chains influence the size of free volumes. They fill them in to a higher extent and decrease their size. A further decrease occurred in case of the series cured with pure sulfur S_8_. Those crosslinked with TBzTD and Vulcuren^®^ exhibited very similar dependence to sulfur-cured samples. The reduction of free volume size with the increase in crosslink density proceeded for MBTS/S_8_ series. Finally, the most evident drop occurred for the ZDT/S_8_ cured samples. The most rapid decrease in the measured structural parameter occurs therefore in case of curing systems, in which accelerators have bulky, inflexible moieties. When the crosslink structure is considered, it would be expected that the short crosslinks reduce the free volume size significantly. Consequently, the longer crosslinks, such as disulfidic and polysulfidic, should result in larger size of free volume. However, the present results show the opposite dependencies. In case of DCP, TMTD, and TBzTD cured samples, the free volume size is larger than for the other samples with longer crosslinks. This proves that the crosslink structure does not influence the free volume size.

For all the investigated series of cured samples, the decrease in free volume size is caused by the presence of crosslinks. They reduce the size of the unoccupied area between the polymer chains. The denser the network is, the smaller the free volumes are. Additionally, differences between particular series of samples indicate that the type of curatives greatly contribute to the observed changes. Part of the curatives grafted on the chains and filled up the free volumes. The higher the bulkiness of the modifications is, the more area is occupied and the free volume is reduced.

The presence of intermolecular crosslinks and modifications of the chains manifests itself via the studied properties. Among others, they influence the density of the samples, reduce the free volume size and stiffen the polymer chains. The present results stay in a good agreement with the theoretical predictions [17]. According to them, in crosslinked polymers the increase in *T*_g_ is followed by a decrease in the free volume size. This is due to the fact that crosslinks strongly connect the polymer chains and lead to a more compact material. 

The measured density of the samples, size of free volumes, and the *T*_g_ values, provide complementary information. They show the effect of various types of curatives on the structural parameters and network microstructure. These structural features are influenced to a great extent by the size of the curatives’ molecules. Due to that, the microstructure of cured elastomers can be predicted and basic structure-properties relations can be established.

The chains’ modifications, “detected” by *T*_g_ and the size of free volumes, possibly affects also other properties of the studied materials, such as e.g., thermal stability and mechanical properties. Studies related to these issues will be a subject to our succeeding paper [23].

## 4. Conclusions

The present research reveals that the crosslink density and the crosslink structures characterize the elastomer network insufficiently. It is also the type and amount of the curatives that influence the microstructure and behavior of the polymers. In research, it is often overlooked that part of the curatives graft on the polymer chains. They do not connect the chains via an intermolecular crosslink, but form modifications. These modifications can be in a form of (i) pendant groups, as fragments of curatives, i.e., accelerators, sulfur, accelerator/sulfur complexes; or (ii) sulfur chains connected to different positions on the same polymer chain, forming cyclic structures. It is proved that besides the elastically-effective crosslinks, the modifications also influence the network parameters and the resulting properties. PALS measurements revealed that the free volume size is dependent on the crosslink density and the type of curatives used. Curatives with a high molecular weight and larger size form bulky and inflexible modifications. They significantly reduce the free volume size. Density measurements showed that the larger the molecules of the curatives are, the denser the packing of cured polymer chains is. This confirms that the curatives locate themselves in free volumes and cause a decrease in their size. The presence of the modifications manifests itself via stiffening of the chains and hindering their molecular mobility. This was proved by following the changes of the glass transition temperature with the use of DSC and DMA. The measurements showed that curatives with a large molecular weight, and present in a considerable amount, caused a significant increase in *T*_g_. In view of the results of this study, a straightforward way to correlate the curatives’ structure with properties is proposed. It is based on an evaluation of the curative size and structure. Furthermore, the presence of the chains’ modifications can be proved by DMA and PALS. For this, these techniques can be used independently or combined. Finally, a strong recommendation is made to express the amount of curatives in the number of moles, instead of phr. This will help to study the microstructure and resulting properties of elastomers in a more reliable way. 

## Figures and Tables

**Table 1:** Characterization of the curatives used in the experiments.

Name (Purity; Producer)	Structural Formula	Molecular Formula, Molecular Weight (g/mol)
**MBTS** 2,2’-dibenzothiazyl disulfide (94%; Arlanxeo, Maastricht, The Netherlands)	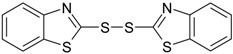	C_14_H_8_N_2_S_4_ 332.49
**DPG** 1,3-diphenylguanidine (97%; Arlanxeo, Maastricht, The Netherlands)	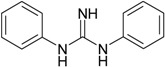	C_13_H_13_N_3_ 211.26
**CBS** *N*-cyclohexyl-1-benzothiazyl sulfenamide (95%; Arlanxeo, Maastricht, The Netherlands)	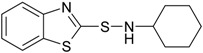	C_13_H_16_N_2_S_2_ 264.41
**TMTD** Tetramethylthiuram disulfide (96%; Arlanxeo, Maastricht, The Netherlands)	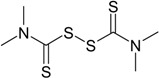	C_6_H_12_N_2_S_4_ 240.43
**TBzTD** Tetrabenzylthiuram disulfide (70%; Shandong Yanggu Huatai Chemical, Yanggu, China)	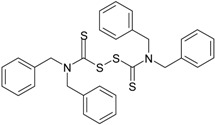	C_30_H_28_N_2_S_4_ 544.82
**Vulcuren^®^** 1,6-bis(*N*,*N*′-dibenzylthio-carbamoyl-dithio)-hexane (87%; Arlanxeo, Maastricht, The Netherlands)	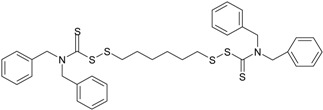	C_36_H_40_N_2_S_6_ 693.11
**ZDT** 2-ethylhexyl zinc dithio-phosphate [13] (50%; Arlanxeo, Maastricht, The Netherlands)	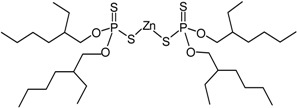	C_32_H_68_O_4_P_2_S_4_Zn 772.47
**DCP** Dicumyl peroxide (98%; Merck Schuchardt, Hohenbrunn, Germany)	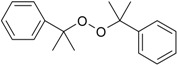	C_18_H_22_O_2_ 270.37
**S_8_** Rhombic sulfur (99.9%; Siarkopol Tarnobrzeg, Tarnobrzeg, Poland)	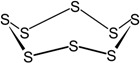	S_8_ 256.52

**Table 2:** Composition of studied samples, given for 100 phr of E-SBR, 5 phr of zinc oxide, and 1 phr of stearic acid.

Sample Name	Amount of Curatives Added (phr)					
Ref.	0.0					
DCP	0.35	0.60	0.75	1.20	2.20	-
TMTD	1.5	3.0	4.5	5.5	7.0	10.0
TBzTD	2.5	5.0	8.0	11.0	15.0	22.0
Vulcuren^®^	3.0	5.0	7.0	9.0	11.0	18.0
S_8_	1.0	2.0	3.5	4.2	5.0	6.5
DPG/S_8_	1.5/1.5	2.0/2.0	2.5/2.5	3.0/3.0	3.6/3.6	4.1/4.1
CBS/S_8_	0.7/0.7	1.2/1.2	1.8/1.8	2.4/2.4	3.0/3.0	3.4/3.4
MBTS/S_8_	0.8/0.8	1.5/1.5	2.0/2.0	3.0/3.0	4.0/4.0	4.4/4.4
ZDT/S_8_	1.5/1.5	2.2/2.2	3.0/3.0	5.0/5.0	6.5/6.5	-

**Table 3:** Dependence between number of moles of accelerator ^1^ and number of moles of sulfur (Ratio = *n*_accelerator_/*n*_sulfur_).

Sample Name	Ratio (-)
DPG/S_8_	1.18
CBS/S_8_	0.92
MBTS/S_8_	0.73
ZDT/S_8_	0.17

^1^ The number of the accelerators’ moles were calculated for the content of the pure active chemical substance in the commercially-available curatives, as listed in Table 1.
